# Trends in the Epidemiological and Clinical Profile of Paracoccidioidomycosis in the Endemic Area of Rio de Janeiro, Brazil

**DOI:** 10.3390/jof9090946

**Published:** 2023-09-20

**Authors:** Eduardo Mastrangelo Marinho Falcão, Dayvison Francis Saraiva Freitas, Ziadir Francisco Coutinho, Leonardo Pereira Quintella, Mauro de Medeiros Muniz, Rodrigo Almeida-Paes, Rosely Maria Zancopé-Oliveira, Priscila Marques de Macedo, Antonio Carlos Francesconi do Valle

**Affiliations:** 1 Laboratory of Clinical Research on Infectious Dermatology, Evandro Chagas National Institute of Infectious Diseases, Fiocruz, Rio de Janeiro 21040-900, Brazil; dayvison.freitas@ini.fiocruz.br (D.F.S.F.); priscila.marques@ini.fiocruz.br (P.M.d.M.); antonio.valle@ini.fiocruz.br (A.C.F.d.V.); 2 Germano Sinval Faria School Health Center, Sergio Arouca National School of Public Health, Fiocruz, Rio de Janeiro 21040-900, Brazil; ziadir@centroin.com.br; 3 Anatomical Pathology Service, Evandro Chagas National Institute of Infectious Diseases, Fiocruz, Rio de Janeiro 21040-900, Brazil; leonardo.quintella@ini.fiocruz.br; 4 Mycology Laboratory, Evandro Chagas National Institute of Infectious Diseases, Fiocruz, Rio de Janeiro 21040-900, Brazil; mauro.muniz@ini.fiocruz.br (M.d.M.M.); rodrigo.paes@ini.fiocruz.br (R.A.-P.); rosely.zancope@ini.fiocruz.br (R.M.Z.-O.)

**Keywords:** paracoccidioidomycosis, neglected diseases, *Paracoccidioides*, mycoses

## Abstract

Paracoccidioidomycosis (PCM) is a neglected endemic mycosis in Latin America. Most cases occur in Brazil. It is classified as PCM infection and PCM disease and is subdivided into chronic (adult type) or acute (juvenile type) disease, with the latter being less frequent and more severe. In 2016, we reported an increase in the numbers of patients diagnosed with acute PCM after a highway’s construction. We conducted a study at INI-Fiocruz, a reference center for infectious diseases, including endemic mycoses, in Rio de Janeiro, Brazil, aiming to deepen the analysis of this new clinical and epidemiological profile of PCM. The authors developed a retrospective study including 170 patients diagnosed with PCM between 2010 and 2019. There was an increase in the number of atypical and severe forms, starting in 2014. In subsequent years, we detected a higher incidence of adverse outcomes with patients requiring more hospitalizations and an increased mortality rate. We estimate that PCM has become more severe throughout the Rio de Janeiro state, affecting a greater number of young individuals and leading to a greater number of and longer hospitalizations. Surveillance measures and close monitoring of future notification data in the state, with emphasis on children, adolescents, and young adults are necessary for a better understanding of the perpetuation of this public health challenge.

## 1. Introduction

Paracoccidioidomycosis (PCM) is a neglected mycosis endemic to Latin America. Most cases occur in Brazil. It is caused by the inhalation of infective propagule. Five species of the genus *Paracoccidioides* are described as agents of the disease (*P. brasiliensis sensu stricto, P. americana, P. restrepiensis, P. venezuelensis,* and *P. lutzii*) [1].

The disease has a spectrum of clinical manifestations associated with the host’s immune response. It is classified as PCM infection (subclinical form) and PCM disease and is subdivided into acute/subacute and chronic forms. The chronic form is the most frequent, usually affects adults, mainly rural workers, and can occur years after exposure to environmental sources of infection. The acute form usually occurs in young people without epidemiological risk activities and, in most cases, is more severe [1,2]. The PCM clinical manifestations also differ between the two forms of presentation. The chronic form usually involves the lungs and the upper aerodigestive tract mucosa, while the acute form affects organs of the phagocytic mononuclear system [3].

The Rio de Janeiro state has long been recognized as an important endemic region for PCM in Brazil [4]. The Evandro Chagas National Institute of Infectious Diseases (INI), Oswaldo Cruz Foundation (Fiocruz) serves as a prominent reference center for PCM clinical care and research, and it is the second leading hospital in Brazil for PCM hospitalizations [5]. The institute’s historical series on this mycosis has been systematically documented since the 1940s. Between 1949 and 1961, the mycologist and dermatologist José Lisboa Miranda and the pulmonologist Machado Filho published 394 cases of PCM from Rio de Janeiro state, and among these cases, 100 were from the INI-Fiocruz [6]. Subsequently, from 1960 to 1986, one of the largest series of PCM cases in Brazil was published, involving 500 cases, with 7.0% of these cases presenting the acute form [7]. Between 1987 and 2009, an additional 460 patient cases were reported, with 10.2% of these cases presenting the acute form [8]. Historically, the chronic form of PCM corresponded to approximately 90% of PCM cases at the INI-Fiocruz. In 2016, an increase in the number of young patients diagnosed with acute PCM at INI-Fiocruz was detected, corresponding to eight (50.0%) cases [9].

In the context of the emergence of acute PCM at our center, we estimated an incidence rate of 8.25 cases of acute PCM per 1 million inhabitants for the state of Rio de Janeiro, which was notably higher than that estimated for its clinical form (1.29/1 million inhabitants). In the period between 2008 and 2014, there was a construction of a large highway in the metropolitan region of Rio de Janeiro, and, interestingly, the patients diagnosed with acute PCM at our center at that time were found to reside between 0.1 and 16.6 km from the construction sites, suggesting a spatiotemporal relationship between the road construction and the emergence of acute PCM cases [9]. It is noteworthy that the epicenter of the cases previously reported experienced the most intense construction activities around 2014, two years before the detection of the outbreak. This finding coincides with a similar timeframe previously reported between the occurrence of a climatic event and the identification of a cluster of acute PCM cases in the hyperendemic region of Botucatu, in São Paulo state, Brazil, suggesting an incubation period of about 24 months for acute PCM [10].

In the present study, the authors aimed to conduct an in-depth analysis of the data during this period of epidemiological PCM transition regarding the clinical aspects of patients diagnosed with PCM at INI-Fiocruz, a reference center in the region where the emergence of acute PCM has been noticed.

## 2. Materials and Methods

This is a retrospective cohort study conducted at the PCM outpatient clinic of INI-Fiocruz. The institutional Research Ethics Committee has approved this study (approval number: 26066619.0.0000.5262). The medical records of patients were reviewed and anonymized to protect patients’ privacy.

All patients admitted at INI-Fiocruz from January 2010 to December 2019 who received the PCM diagnosis according to the II Brazilian Consensus on PCM were included in the study. Briefly, diagnostic criteria included clinical manifestations compatible with PCM, and the presence of fungal elements suggestive of *Paracoccidioides* sp. in any clinical specimen and/or compatible clinical manifestations with detectable anti-*Paracoccidioides* antibodies in serum samples using immunodiffusion (ID) performed with a pool of crude antigens was obtained from isolates Pb01 and Pb339. Patients were clinically classified as having the chronic or acute form according to their symptoms, affected organs, and natural history of the disease [3].

The variables analyzed included gender, age, skin color, place of residence, epidemiological risk activities, smoking, alcoholism, comorbidities, affected organs, PCM clinical form, PCM specific serology data, mycological and histopathological examination of clinical samples, medications prescribed to treat PCM, treatment time, number of hospitalizations, and clinical outcomes. By analyzing these variables comprehensively, we aimed to uncover significant patterns and associations, providing valuable data for a deeper understanding of PCM during this period of epidemiological transition at our reference center.

Statistical analyses were performed using the R program version 4.3.0, considering a significance level of 5%. Frequencies, means, and medians were calculated to describe the variables under investigation. To determine the associations and differences between categorical variables, Fisher exact test or Chi-square test, whichever appropriate, was used. For continuous variables or ordinal data, the Wilcoxon test was performed to test whether the two independent groups differ significantly from each other.

In order to address the research gaps related to deaths or hospitalization data in this study, the public databases of Mortality Information System (SIM) and Hospitalization Information System (SIH/SUS) of the Ministry of Health of Brazil [11] were also accessed, respectively.

## 3. Results

**Figure 1:**
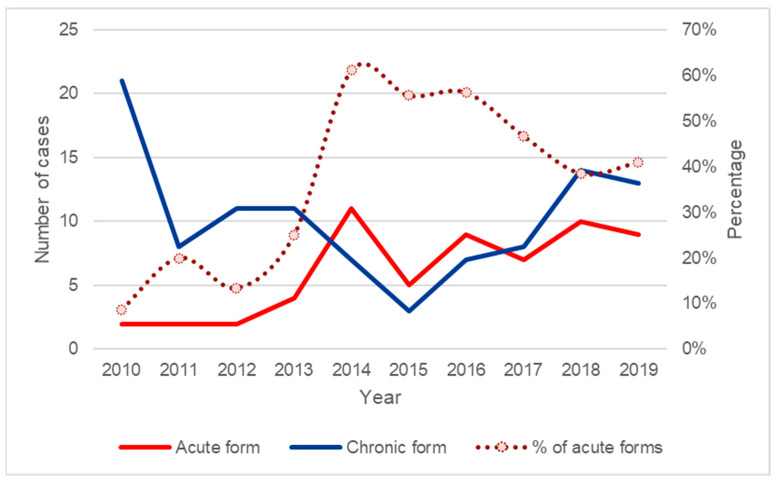
Frequencies of paracoccidioidomycosis clinical forms diagnosed at INI-Fiocruz (2010–2019). Source: INI electronic medical record system (Sipec).

We included 170 patients diagnosed with PCM during the period of the study. Based on the frequencies of both PCM clinical forms identified each year (Figure 1) and the period of construction of the highway related to the previously described outbreak [9], we identified 2014 as the year when acute cases effectively started to present an absolute frequency above the annual average. Thus, we divided the patients included in this study in two groups and compared the variables of interest between them: group A patients diagnosed with PCM between 2010 and 2013, and group B patients diagnosed with PCM between 2014 and 2019.

Between 2010 and 2013, 64 patients were diagnosed with PCM at INI-Fiocruz, an average of 16 cases/year, while 106 were diagnosed between 2014 and 2019, with a similar average of 17.7 cases/year.

### 3.1. Demographic and Epidemiological Aspects

Concerning demographic data, group A presented 54 (84.4%) male patients, and the median age was 49 years. Group B had 78 (73.6%) males, and the median age was 42 years. Comparing the two groups, the difference in gender distribution and age group was not statistically significant. The place of residence (metropolitan versus other regions) was similar in both groups. Regarding risk activities for PCM infection (contact with rural activity and/or armadillo hunting), in group A, 45 (70.3%) patients reported a positive epidemiological history, and in group B, 52 (49.1%) patients reported a positive epidemiological history. Group A presented 48 (75.0%) smokers, and group B represented 57 (53.8%) smokers. The difference was statistically significant for both epidemiological and smoking histories (Table 1).

### 3.2. Clinical Aspects

Regarding the PCM clinical forms, group A presented 53 (82.8%) patients with chronic PCM and 11 (17.2%) patients with acute PCM. In group B, 52 (49.1%) presented the chronic form and 54 (50.9%) had the acute.

**Figure 2:**
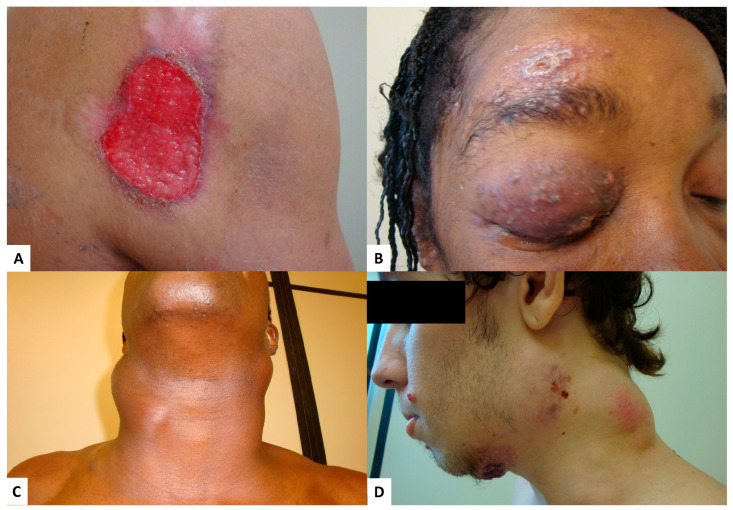
Paracoccidioidomycosis patients diagnosed at INI-Fiocruz between 2014 and 2019: (**A**) extensive ulcer on the right shoulder; (**B**) ulcero-crusted lesion in the frontal region surrounded by papules and erythema with eyelid edema; (**C**) enlarged lymph nodes in the cervical region; and (**D**) enlarged lymph nodes in the cervical region with some fistulization of the skin.

The main organs affected in group A were the lungs, the oral mucosa, and the skin, while in group B, there was a predominant involvement of lymph nodes, lungs, and skin (Figure 2A,B).

There was a statistically significant difference observed for lymph node involvement (Table 2). In group B, eight (7.5%) patients had large and/or confluent lymph nodes in the form of large tumors (Figure 2C,D), reaching up to 6 cm in diameter, and one of them had an extrinsic compression of the biliary tract, requiring hospitalization and treatment with amphotericin B.

While in the first period no patient had PCM in the biliary tract, in the second period, three patients had clinical manifestations resembling cholangiocarcinoma due to involvement of the biliary tract, and one of them required a cholecystectomy with partial gastrectomy and complex reconstruction before admission to the INI-Fiocruz and resulted in death from sepsis after 24 months of treatment. The other two patients were cured (one of them with portal hypertension as a sequela).

In one patient, in group B, the stomach was involved. The intestine was affected in six (5.7%) patients. One of them had clinical manifestations like Crohn’s disease, and immunosuppression was initiated before admission to our service, which led to the dissemination of PCM. Another patient from group B had the esophagus affected due to fistulization of mediastinal lymph nodes.

In group A, the skin lesions were ulcerated in 92.3% of cases, restricted to the face and scalp in 50.0% and disseminated in 11.5% of cases. In group B, skin lesions were ulcerated in 72.2%, restricted to the face and scalp in 31.5%, and disseminated in 48.1% of cases.

Two patients presented signs and symptoms suggestive of ocular involvement in the second period of the study (Figure 2B). The first, in addition to skin lesions on the face, presented with lymph node enlargement and weight loss and had dacryocystitis that resolved along with the other symptoms, requiring 24 months of treatment. The second presented with visual blurring concomitant with skin lesions and lymphadenopathy, and was ultimately cured, requiring 25 months of treatment.

In addition, group B had more atypical clinical presentations such as portal hypertension in three patients (2.7%) and gastric PCM in one patient (0.9%). One patient with involvement of lymph nodes and hepatosplenomegaly presented with a dramatic worsening of the condition, leading to septic shock and death. The direct microscopic examination of a blood culture with Gram staining revealed *Paracoccidioides* sp. yeast-like cells.

Regarding comorbidities, we detected six patients living with HIV (PLWHA) in both group A (9.4%) and group B (5.7%). Other forms of immunosuppression occurred exclusively in group B and included the use of corticosteroids (three patients), leukemia (one patient), and the use of methotrexate (two patients).

A 90-year-old patient with chronic lymphocytic leukemia presented with clinical manifestations like the acute form of PCM, required hospitalization twice, and abandoned treatment after 12 months. Another patient, using methotrexate due to a diagnosis of Sjogren’s syndrome, had a pelvic abscess along with pleural and bone involvement resulting from PCM, and was clinically cured after 19 months of treatment.

### 3.3. Diagnostic Methods

**Figure 3:**
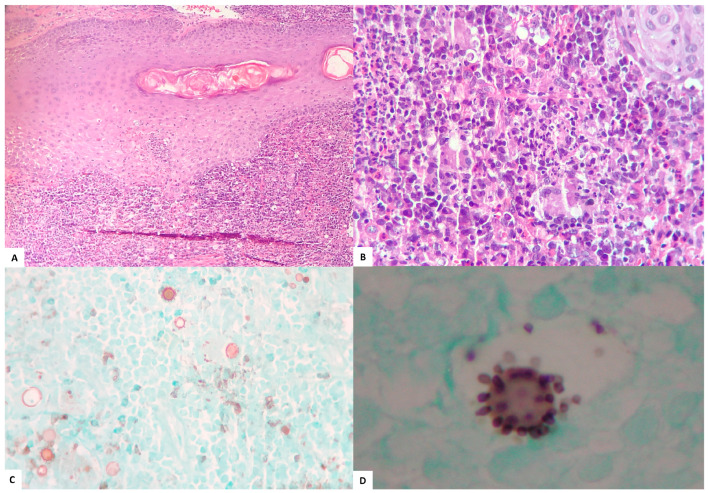
Anatomopathological examination of skin fragments from patients diagnosed at INI-Fiocruz between 2010 and 2019: (**A**) HE ×100, diffuse granulomatous dermatitis with giant cells, some large round and refractile fungal cells, and infiltration of follicular epithelium by neutrophils; (**B**) HE ×400, diffuse suppurative granulomatous dermatitis with giant cells, plasma cells, and eosinophils, and some large and small, round, and refractile fungal cells; (**C**) Grocott ×400, fungal forms with budding in a “steering wheel” pattern.; and (**D**) Grocott ×1000 (immersion), fungal form with budding in a “steering wheel” pattern.

The main methods employed to diagnose PCM are summarized in Table 3. The diagnosis was proven by the visualization of fungal structures in 165 (97.1%) patients. Meanwhile, five (2.9%) patients were diagnosed serologically. Histopathological examination, performed for 127 (74.7%) patients, was the main method used to confirm PCM diagnosis, showing the presence of multiple budding fungal structures (Figure 3) in 122/127 (96.1%) cases. The mycological examination was performed for 74 (43.5%) patients, detecting these structures in 40/74 (54.1%) cases. In group A, the histopathology was confirmatory for PCM for 47 (73.4%), direct mycological examination for 9 (14.1%), and culture for 10 (15.6%) patients. In group B, the histopathology was confirmatory for PCM in 75 (70.8%), direct mycological examination in 31 (29.2%), and culture for 34 (32.1%) patients.

It was not possible to obtain isolates from the patients of the study, but some species had already been previously identified in another study. Among the patients from group A, it was possible to identify six (9.3%) strains yielded from positive cultures, all identified as *P. brasiliensis*. Among the patients from group B, it was possible to identify seven (6.6%) strains, six as *P. brasiliensis* and one (0.9%) as *P. americana*.

Concerning the serological diagnosis of PCM, it was positive in 51 (79.7%) patients of group A, with titers reaching at least 1:512 in 2 (3.1%) patients. Positivity of serology among patients with the acute form was 81.8%, while in patients with the chronic form, it was 79.2%.

In group B, 83 (78.3%) patients had positive results, with titers reaching at least 1:512 in 11 (10.4%) patients. Positivity of serology among patients with the acute form was 70.4%, while in patients with the chronic form, it was 86.5%.

### 3.4. Therapeutic Features

Itraconazole (ITZ) was the main drug prescribed to treat PCM in both groups (Table 3). Group A included 51 (79.7%) patients receiving ITZ, 27 (42.2%) using trimethoprim-sulfamethoxazole (TMP-SMZ), and 9 (14.1%) receiving amphotericin B (AMB) for intravenous therapeutic induction. Among the patients with the acute form, 10 (90.9%) received ITZ, 6 (54.5%) TMP-SMZ, and 7 (63.6%) AMB. Among the patients with the chronic form, 41 (77.4%) received ITZ, 21 (39.6%) TMP-SMZ, and 2 (3.8%) AMB.

Group B had 87 (82.1%) patients on ITZ, 49 (46.2%) on TMP-SMZ, and 44 (41.5%) on AMB. Among the patients with the acute form, 44 (81.5%) received ITZ, 22 (40.7%) TMP-SMZ, and 37 (68.5%) AMB. Among the patients with the chronic form, 43 (82.7%) received ITZ, 27 (51.9%) TMP-SMZ, and 7 (13.5%) AMB.

There was a statistically significant difference for AMB between group A and B (*p*-value = 0.001).

The median treatment time in group A was 18.5 months (ranging from 6.7 to 68.1 months), reaching 25.4 (6.7 to 68.1) months in patients with the acute form. In group B, it was 24.9 months (ranging from 10.0 to 50.7 months), reaching 25.2 (10.0 to 50.7) months in patients with the acute form. There was a statistically significant difference regarding the treatment time between the two periods of the study (*p*-value = 0.02).

### 3.5. Prognostic Data

Twelve (18.8%) patients of group A required one hospitalization. In group B, 44 (41.5%) patients required hospitalization, being one single hospitalization for 35 patients, two hospitalizations for 6, and three hospitalizations for 3 patients (Table 3). The calculated odds ratio (OR) between the two periods of hospitalizations was 3.03 (95% CI: 1.48–6.59, *p*-value = 0.002). Among the patients hospitalized in group A, one (1.6%) were admitted into an intensive care unit (ICU), and in group B, six (5.7%) patients were admitted into an ICU.

One death (1.6%) of a patient with the chronic form of PCM due to an unknown cause was recorded in group A. In group B, seven (6.6%) patients died, five with the acute form of PCM. Two died due to unknown causes and one due to external causes. In four deaths, PCM was the underlying cause. The difference between periods was not statistically significant.

## 4. Discussion

Most patients infected with *Paracoccidioides* sp. are asymptomatic. Among those who develop the disease, 75–95% develop the chronic form [1,2]. Historically, at the INI-Fiocruz, the acute form of the disease has varied between 7.0 and 10.0% [7,8]. In the present study, a progressive increase in the percentage of patients diagnosed with the acute form of the disease was observed, resulting in an up to 37.0% increase in the period between 2010 and 2019 and a 50.9% increase in the period between 2014 and 2019. In 2014, the percentage was 61.1%, and in the last year of the study, this rate remained high (40.9%). This increase to levels higher than those reported in the literature occurred in a period that encompasses the previously described outbreak of the acute form of the disease probably associated with the construction of the “Raphael de Almeida Magalhães” highway [9]. These levels are the highest ever recorded for this clinical form in Brazil, much higher than those reported in other hyperendemic regions of Brazil, such as Botucatu, São Paulo state [10]. In addition, a reduction in the frequency of PCM chronic form has been probably occurring over the years in stabilized endemic areas, due to changes in the rural environment such as mechanization reducing the workers’ exposure to *Paracoccidioides* spp. [4].

Environmental sources close to the mentioned highway were investigated after the outbreak report. Genetic material of *P. brasiliensis* was detected in soil samples [12], reinforcing the hypothesis that there is a correlation between the construction and the increase in the number of acute cases. Furthermore, other health services also reported severe and atypical cases of the acute form of the disease in the metropolitan area of the state [13].

Considering the need to improve the epidemiological surveillance of systemic mycoses and their interaction with the environment, a relevant issue that still has low visibility, the State Department of Health of Rio de Janeiro state (SESRJ) published a resolution in 2021 making PCM a notifiable disease, along with other systemic mycoses in this region [14]. This measure will provide more accurate data on these diseases, in addition to the progressive structuring of sentinel services. Until April 2023, according to data provided by SESRJ, 86 cases of PCM were reported by health units in the state of Rio de Janeiro, with 40.7% of these cases being from the INI-Fiocruz [15]. This institute was the health unit with the most notified cases besides receiving referred patients previously notified by other services. Therefore, the number of patients treated at this institute represents most cases in the state. Among these new notified cases, 13.9% patients were under 18 years old, an age group compatible with the acute form of PCM [15]. Considering that the INI/Fiocruz does not treat children, we estimate that the occurrence of acute forms may be higher than that herein reported.

In addition to an increase in the occurrence of acute forms, in the second period herein studied, changes were observed in the clinical and epidemiological characteristics of the disease in relation to what was observed in the first period and reported in the literature [1,2,3,7,16]. Between 2014 and 2019, there was a lower median age, lower percentages of patients with history of contact with rural activities or hunting, and of smokers diagnosed with PCM. In addition to possible epidemiological changes related to reduced exposure to rural work [7] or the worldwide reduction in smoking, these characteristics are consistent with the acute form of the disease [1] and reflect an increase in the number of cases.

From a clinical point of view, in the chronic form, most often patients present with pulmonary and mucosa of the upper aerodigestive tract’s involvement, while patients with the acute form of PCM more frequently have their reticuloendothelial system affected with the disease [1]. The most affected organs were the lung, mucosa of the upper aerodigestive tract, lymph nodes, and skin in both periods. However, there was a statistically significant increase in the percentage of patients with lymph node involvement (*p*-value = 0.003), compatible with an increase in the acute form.

Confluent lymph nodes can lead to large masses/tumors that can compress vital structures [1,2]. Eight (7.5%) patients in the second study period had these large tumors demonstrating a higher severity of cases, which can lead to an increase in the number of hospitalizations.

In addition to extrinsic compression, the biliary ducts can also be directly affected by PCM. This manifestation is rare and can simulate neoplasia [17]. All cases observed in the second period simulated a cholangiocarcinoma. It is necessary to emphasize the importance of considering PCM as a differential diagnosis in endemic areas to reduce the progression to severe sequelae and minimize the need for extensive surgical interventions.

Hepatic involvement can occur due to cholestasis or direct involvement of the fungus in the hepatic parenchyma and can be potentially fatal. Although frequent, most of the times, it is asymptomatic [1]. In cases with more fungal burden, portal/periportal fibrosis was suggested to be more frequent [18]. All three patients in the second period of the study, who had portal hypertension, had a disseminated and a more severe form of the disease.

The skin is an organ frequently affected in PCM, via hematogenous route or adjacent to mucosal lesions or lymph nodes. Lesions are pleomorphic and occur most frequently on the face [1]. In the period from 2010 to 2013, most skin lesions were ulcers, in a small number and restricted to the head. In the second period, 54 (50.9%) patient had skin involvement, and a higher percentage of disseminated lesions was observed, also affecting the trunk and limbs in addition to the head. They were polymorphic, ranging from ulcers to molluscum contagiosum-like papules.

Neuroparacoccidioidomycosis is a rare and severe form of PCM. Reports of central nervous system (CNS) involvement in PCM are more frequent in patients with the chronic and disseminated form of the disease, and it more frequently presents as mass-like intracerebral lesions [19,20]. When comparing the two periods of the study, the proportion was similar (1.6% versus 1.9%), despite the increase in acute forms. This might be associated with a higher chance of dissemination due to a delay in diagnosis, since the acute form is rare and unknown by professionals in basic health units [10,21].

Another vital organ affected by PCM is the adrenal glands, leading to more severe forms of the disease. The involvement of these glands can be as high as 80.0% [1,3]. PCM is the second cause of primary adrenal insufficiency in Brazil, just behind autoimmune etiology [22]. Among the patients in our series, 15.3% had some involvement of these glands, with an increased activity, although not significant, in the second period (9.4 versus 18.9%), reiterating the greater severity of patient conditions between 2014 and 2019. In both periods, for some time, there were shortages of tests, which may have resulted in underdiagnosis in patients without symptoms of adrenal insufficiency.

Gastrointestinal PCM is rarely diagnosed because of nonspecific symptoms. The intestines are affected more frequently than the stomach and esophagus [23]. Misdiagnosis of Crohn’s disease and the use of immunosuppressants can lead to a worsening of the condition, as observed in one of the reported patients [24,25].

The eyes are rarely affected by PCM. There are reports in the literature of cases involving the eyelids, conjunctiva [26], choroid, retina, and optic disc [27]. Cases of ocular involvement occurred only in the second period, probably due to the higher number of cases of more disseminated disease.

In immunosuppressed patients, the diagnosis can be challenging due to atypical clinical manifestations and impaired serological diagnosis [28]. HIV infection can alter the natural history of PCM and recently there has been an increase in the number of coinfection cases in Latin America [29]. When comparing the two studied periods, there was no significant increase in the percentage of PLWHA, suggesting that even with the apparent urbanization of PCM, this vulnerable population was not significantly affected.

Among the reported cases, other forms of immunosuppression were observed (use of corticosteroids, leukemia, and use of methotrexate). There was a greater number of immunosuppressed patients in the second period of the study, possibly because of the urbanization of PCM in the state.

Confirmatory diagnosis of PCM requires the visualization of the fungus using mycological or histopathological examination [3]. The histopathological examination with hematoxylin–eosin stain also allows the evaluation of the host’s immune response [1]. The histopathological examination was the main exam in the diagnosis of patients in both periods of the study. Although mycological direct examination is faster and less expensive, it was not always performed before referring patients to the INI-Fiocruz. In these patients, PCM was not considered as a possible diagnosis at the time of biopsy.

The ID has been the serological test of choice for the diagnosis and therapeutic follow-up of PCM. Its specificity and sensitivity can vary from 65–100% according to the antigen used and the *Paracoccidioides* species causing the infection [1]. Higher ID titles have already been reported in the literature associated with the acute form of PCM and more severe forms of the disease [30]. In the present study, a negative ID was observed in 18.8% of the total number of patients. In the second period of the study, the percentage of false negative tests was higher (20.8%), as well as the occurrence of titers greater than 1:512 (10.4%). Several reasons for an increase in false negative results of the acute form were proposed [30], and we believe the formation of immune complexes, which occludes the immunoglobulin paratope, or the presence of asymmetric antibodies could be found in those patients’ sera.

Currently, no differences between the clinical manifestations of the *Paracoccidioides* species have been demonstrated [31]. Although the present study does not include the molecular identification of the species, some samples were part of another previously published study [31]. Although these samples represent a small percentage of the total number of cases, it is not yet possible to state whether the epidemiological pattern of species distribution in the state has changed during the study period. In the previously published study [31], of the 47 isolates identified, 6 corresponded to *P. americana* identified between 2002 and 2008, all associated with chronic forms, while an isolate of *P. americana* corresponding to a patient with the acute form of the outbreak was identified in 2016.

PCM treatment is long, requiring at least 9 months of medication. Itraconazole is the drug of choice, while amphotericin B is reserved for severe and disseminated forms, as a therapeutic induction, until clinical stability is achieved [3]. The most used drug for the treatment of PCM throughout the study period was itraconazole (81.2%). There was a greater use of amphotericin B for the treatment of patients in the second study period (14.1% versus 41.5%, *p*-value = 0.001). In the period from 2014 to 2019, the treatment time was also longer (*p*-value = 0.02). These data reflect an increase in more severe forms of the disease in the second period of the study.

The increase in the percentage of acute forms also led to a greater number of hospitalizations (*p*-value = 0.003). Patients in the second period of the study were more likely to be hospitalized than those in the first period (OR = 3.03, 95% CI: 1.48–6.59, *p*-value = 0.002). Despite a higher percentage of patients requiring ICU in the second period, there was no statistically significant difference.

Poor adherence is a relevant issue in the treatment of patients with PCM. Prolonged treatment, distance from the health unit, and initial improvement in clinical manifestations lead patients to miss appointments and use medications incorrectly [32]. Although statistically insignificant (*p*-value *=* 0.453), the percentage of abandonment in the first period of the study was slightly higher (21.9 versus 16.0) probably due to the more severe manifestations of the disease and the change in the profile of affected patients in the second period. Poor adherence also led some patients to require a longer treatment period.

There were limitations inherent to the characteristics of a retrospective study and to the social situation of the individuals participating in the research. Some tests were not performed, such as the parasitological examination of feces, which may have impacted some undiagnosed cases of strongyloidiasis, a disease that is often associated with chronic forms of PCM [1]. As this is a unicentric study and does not include a pediatric population, there is a limitation in generalizing the cases to the entire population. In addition, some data were not obtained due to the lack of registration, i.e., the absence of medical records.

The geographical spread of PCM and emergence of its acute forms are challenging to control as they depend on several environmental and anthropogenic factors [4,9,10,33]. Monitoring new cases of the disease [14] and the development of research for new therapeutic and prophylactic protocols [34] are essential.

## 5. Conclusions

It is possible to conclude that there was a change in the clinical and epidemiological profile of PCM in the main reference center for this disease in the state of Rio de Janeiro, starting in 2014. Patients with atypical and severe forms were admitted more frequently, requiring more hospitalizations and resulting more frequently in deaths. It is estimated that, throughout the state, the disease has become more severe, affecting a greater number of young individuals, with the acute form of the disease, and leading to a greater number of and longer hospitalizations. Surveillance measures and analysis of future notification data in the state and across the country, with an emphasis on children, adolescents, and young adults, are necessary for a better understanding of the perpetuation of this event.

## Figures and Tables

**Table 1:** Epidemiological data of the patients with paracoccidioidomycosis from INI-Fiocruz (2010–2019).

	Total *n* (%)	Group A (2010–2013) *n* (%)	Group B (2014–2019) *n* (%)	*p*-Value
**Gender**				0.148
Male	132 (77.6)	54 (84.4)	78 (73.6)	
Female	38 (22.4)	10 (15.6)	28 (26.4)	
**Age group**				0.129
<18	7 (4.1)	3 (4.7)	4 (3.8)	
18–30	40 (23.5)	8 (12.5)	32 (30.2)	
31–45	32 (18.8)	13 (20.3)	19 (17.9)	
46–59	60 (35.3)	27 (42.2)	33 (31.1)	
>60	31 (18.2)	13 (20.3)	18 (17.0)	
**Skin color**				0.126
White	49 (28.8)	25 (39.1)	24 (22.6)	
Black	37 (21.8)	12 (18.8)	25 (23.6)	
Mixed	77 (43.0)	27 (42.2)	50 (47.2)	
Uninformed	7 (4.1)	0 (0.0)	7 (6.6)	
**Residence**				0.987
Metropolitan	117 (68.8)	44 (68.8)	73 (68.9)	
Countryside	53 (31.2)	20 (31.3)	33 (31.1)	
**Epidemiological history**				0.010
Yes	97 (57.1)	45 (70.3)	52 (49.1)	
No	73 (42.9)	19 (29.7)	54 (50.9)	
**Smoking**				0.009
Yes	105 (61.8)	48 (75.0)	57 (53.8)	
No	65 (38.2)	16 (25.0)	49 (46.2)	
**Alcoholism**				0.560
Yes	50 (29.4)	21 (32.8)	29 (27.4)	
No	120 (70.6)	43 (67.2)	77 (72.6)	

**Table 2:** Clinical data of the patients with paracoccidioidomycosis from INI-Fiocruz (2010–2019).

	Total *n* (%)	Group A (2010–2013) *n* (%)	Group B (2014–2019) *n* (%)	*p*-Value
**Clinical form**				<0.001
Chronic	107 (62.9)	53 (82.8)	52 (49.1)	
Acute	63 (37.1)	11 (17.2)	54 (50.9)	
**Affected organ**				
Lungs	95 (55.9)	35 (54.7)	60 (56.6)	0.932
Lymph node	87 (51.2)	23 (35.9)	64 (60.4)	0.003
Skin	80 (47.0)	26 (40.6)	54 (50.9)	0.251
**Mucosa**				
Oral	73 (42.9)	32 (50.0)	41 (38.7)	0.198
Larynx	35 (20.6)	13 (20.3)	22 (20.8)	0.944
Nasal	21 (12.4)	8 (12.5)	13 (12.3)	0.964
Pharynx	13 (7.6)	8 (12.5)	5 (4.7)	0.120
Trachea	1 (0.6)	0 (0.0)	1 (0.9)	1.000
Penis	1 (0.6)	1 (1.6)	0 (0.0)	0.376
Adrenal	26 (15.3)	6 (9.4)	20 (18.9)	0.101
Spleen	21 (12.4)	4 (6.3)	17 (16.0)	0.150
Liver	15 (8.8)	2 (3.1)	13 (12.3)	0.079
Bone	5 (2.9)	2 (3.1)	3 (2.8)	1.000
CNS	3 (1.8)	1 (1.6)	2 (1.9)	1.000
Intestine	8 (4.7)	2 (3.1)	6 (5.7)	0.711
Biliary ducts	3 (1.8)	0 (0.0)	3 (2.8)	0.291
**Comorbidities**				
SAH	27 (15.9)	9 (14.1)	18 (17.0)	0.773
DM	8 (4.7)	2 (3.1)	6 (5.7)	0.702
COPD	8 (4.7)	4 (6.3)	4 (3.8)	0.476
HIV/AIDS	12 (7.1)	6 (9.4)	6 (5.7)	0.543
Tuberculosis	9 (5.3)	3 (4.7)	6 (5.7)	1.000
Neoplasia	8 (4.7)	1 (1.6)	7 (6.6)	0.261
Strongyloidiasis	11 (6.5)	5 (7.8)	6 (5.7)	0.817

CNS: central nervous system; SAH: systemic arterial hypertension; DM: diabetes mellitus; COPD: chronic obstructive pulmonary disease; and HIV/AIDS: human immunodeficiency virus/acquired immunodeficiency syndrome.

**Table 3:** Diagnostic, therapeutic, and outcome data of the patients with paracoccidioidomycosis from INI-Fiocruz (2010–2019).

	Total *n* (%)	Group A (2010–2013) *n* (%)	Group B (2014–2019) *n* (%)	*p*-Value
**Hospitalizations**				0.003
0	114 (67.1)	52 (81.3)	62 (58.5)	
1	47 (27.6)	12 (18.8)	35 (33.0)	
2	6 (3.5)	0 (0.0)	6 (5.7)	
3	3 (1.8)	0 (0.0)	3 (2.8)	
**Treatment ^1^**				
Itraconazole	138 (81.2)	51 (79.7)	87 (82.1)	0.854
SMX-TMP	76 (44.7)	27 (42.2)	49 (46.2)	0.723
Amphotericin B	53 (31.2)	9 (14.1)	44 (41.5)	0.001
Fluconazole	6 (3.5)	1 (1.6)	5 (4.7)	0.411
Sulfadiazine	3 (1.8)	1 (1.6)	2 (1.9)	1.000
**Laboratorial PCM diagnosis ^1^**				
Histopathology	122 (71.8)	47 (73.4)	75 (70.8)	0.841
Direct examination	40 (23.5)	9 (14.1)	31 (29.2)	0.038
Culture	44 (25.9)	10 (15.6)	34 (32.1)	0.028
**Diagnostic serology**				0.334
1:1–1:4	17 (10.0)	6 (9.4)	11 (10.4)	
1:8–1:32	35 (20.6)	15 (23.4)	20 (18.9)	
1:64–1:256	35 (20.6)	9 (14.0)	26 (24.5)	
≥1:512	13 (7.6)	2 (3.1)	11 (10.4)	
Non reactive	31 (18.2)	9 (14.0)	22 (20.8)	
Reactive, titration not performed	34 (20.0)	19 (29.7)	15 (14.2)	
Not performed	5 (2.9)	4 (6.3)	1 (0.9)	
**Outcome**				
Cure	127 (74.7)	49 (76.6)	78 (73.6)	0.802
Death	8 (4.7)	1 (1.6)	7 (6.6)	0.261
Lost to follow-up	31 (18.2)	14 (21.9)	17 (16.0)	0.453
Under treatment	3 (1.8)	0 (0.0)	3 (2.8)	0.291
Transference ^2^	1 (0.6)	0 (0.0)	1 (0.9)	

^1^ Sum can be greater than 100%, as the same patient can be in more than one category; ^2^ one patient chose to finish the treatment in another institution; SMX-TMP: sulfamethoxazole with trimethoprim; PCM: paracoccidioidomycosis.

## Data Availability

Not applicable.
